# Nano-WSe_2_ Is Absorbable and Transformable by Rice Plants †

**DOI:** 10.3390/molecules27227826

**Published:** 2022-11-13

**Authors:** Xue Tian, Hongxin Xie, Jincheng Li, Liwei Cui, Yong-Liang Yu, Bai Li, Yu-Feng Li

**Affiliations:** 1 Department of Chemistry, College of Sciences, Northeastern University, Shenyang 110819, China; 2 CAS Key Laboratory for Biomedical Effects of Nanomaterials and Nanosafety, CAS-HKU Joint Laboratory of Metallomics on Health and Environment, Beijing Metallomics Facility, National Consortium for Excellence in Metallomics, Institute of High Energy Physics, Chinese Academy of Sciences, Beijing 100049, China; 3 University of Chinese Academy of Sciences, Beijing 100049, China

**Keywords:** nano tungsten selenide, rice, germination, growth, XAS, XRF

## Abstract

As typical transition metal dichalcogenides (TMDC), tungsten selenide (WSe_2_) nanosheets (nano-WSe_2_) are widely used in various fields due to their layered structures and highly tunable electronic and magnetic properties, which results in the unwanted release of tungsten (W) and selenium (Se) into the environment. However, the environmental effects of nano-WSe_2_ in plants are still unclear. Herein, we evaluated the impacts and fate of nano-WSe_2_ and micro-WSe_2_ in rice plants (*Oryza sativa* L.). It was found that both nano-WSe_2_ and micro-WSe_2_ did not affect the germination of rice seeds up to 5000 mg/L but nano-WSe_2_ affected the growth of rice seedlings with shortened root lengths. The uptake and transportation of WSe_2_ was found to be size-dependent. Moreover, W in WSe_2_ was oxidized to tungstate while Se was transformed to selenocysteine, selenomethionine, Se^IV^ and Se^VI^ in the roots of rice when exposed to nano-WSe_2_, suggesting the transformation of nano-WSe_2_ in rice plants. The exposure to nano-WSe_2_ brought lipid peroxidative damage to rice seedlings. However, Se in nano-WSe_2_ did not contribute to the synthesis of glutathione peroxidase (GSH-Px) since the latter did not change when exposed to nano-WSe_2_. This is the first report on the impacts and fate of nano-WSe_2_ in rice plants, which has raised environmental safety concerns about the wide application of TMDCs, such as WSe_2_ nanosheets.

## 1. Introduction

Transition metal dichalcogenides (TMDCs) are a family of 2D nanosheets typically in the form of MX_2_, where M is the transition metal and X is the chalcogen. In recent years, TMDCs have attracted wide attention due to their layered structures and highly tunable electronic and magnetic properties [1]. TMDCs have been applied in the fields of energy [2], environment [3], biomedicine [4] and agriculture [5] due to their excellent physical, chemical, electrical and biological properties [6]. For example, molybdenum sulfide (MoS_2_) nanosheets have been applied in biomedicine, biosensing and bioimaging [7]. It was noticed that Mo in the MoS_2_ nanosheet could be used for the synthesis of molybdenum enzymes in animals [8], suggesting the bioavailability of MoS_2_ in animals. It was also found that MoS_2_ promoted rice growth by regulating chlorophyll synthesis and aquaporin gene expression [9].

Tungsten selenide (WSe_2_) nanosheet (nano-WSe_2_) is another typical TMDC, which has excellent mechanical, optical, electrical and magnetic properties [10]. It is used in photovoltaic devices and ultra-thin LEDs and is an additive in lubricants due to its flake structure with small friction coefficient [11]. The reconfiguration or doping on WSe_2_ with other atoms [12] enhances its applications in environmental engineering [13] and chemical engineering [14]. The wide application of nano-WSe_2_ increases the possibility of its release into the environment. The environmental toxicity of nano-WSe_2_ is still largely unknown. To date, only one study on the cytotoxicity of WSe_2_ has been reported, which found at least 50% cell death at a dose of 400 μg/mL [15].

Tungsten (W) is an essential element for archaea [16], such as *Pyrococcus furiosus* [17], and is linked to organic cofactors, such as tungsten cofactors [18]. It can replace Mo in the molybdenum pterin cofactor to reduce the severity of intestinal inflammation in mice with colitis [19]. However, W is also an environmental pollutant and has been associated with leukemia [20]. The increasing release of W to water and soil has attracted more and more attention since W was identified as an emerging environmental pollutant by the U.S. Environmental Protection Agency [21]. Selenium (Se) is an essential element for animals and human beings but is toxic to plants [22]. The indiscriminate incorporation of seleno-amino acids into proteins can impair protein functions and ultimately lead to physiological malfunction [23]. Se is involved in many enzymatic activities and the regulation of the physiological metabolism. For example, Se can reduce stress injury by down-regulating galactose metabolism and activating the metabolism of glutamate and glutamine [24] and enhances the production of heat shock proteins (HSPs) and glutathionase activity to alleviate heat stress-induced oxidative damage in vivo [25].

Plants are an important part of the ecosystem and play important roles in ecological balance [5]. Rice is one of the staple foods for human beings, which has also been applied as a model plant for ecotoxicological study [26]. The absorption and accumulation of toxic elements by crops will affect the food safety and even bring harm to human health [27]. Considering the low solubility of WSe_2_, it is generally believed that it is not bioavailable to plants; however, it is not clear whether WSe_2_ at nano size is bioavailable and transformable or not. Understanding the bioavailability and phytotoxicity of nano-WSe_2_ on crops is important for food safety and agricultural sustainability but such information is currently lacking.

To address these knowledge gaps, the impact and fate of nano-WSe_2_ in rice plants (*Oryza sativa* L.) was studied using WSe_2_ powder (micro-WSe_2_) for comparison in this study. The impacts on germination rate and growth of rice seedlings by nano-WSe_2_ or micro-WSe_2_ were first studied. Then the biological responses after exposure to nano-WSe_2_ or micro-WSe_2_ were compared. The distribution and transformation of W and Se in different tissues of rice plants were then examined. To the best of our knowledge, this is the first report on the impact and fate of nano-WSe_2_ on rice plants, which may shed light on the safe application of TMDCs.

## 2. Results and Discussion

### 2.1. The Size of Nano-WSe_2_ and Micro-WSe_2_

**Figure 1:**
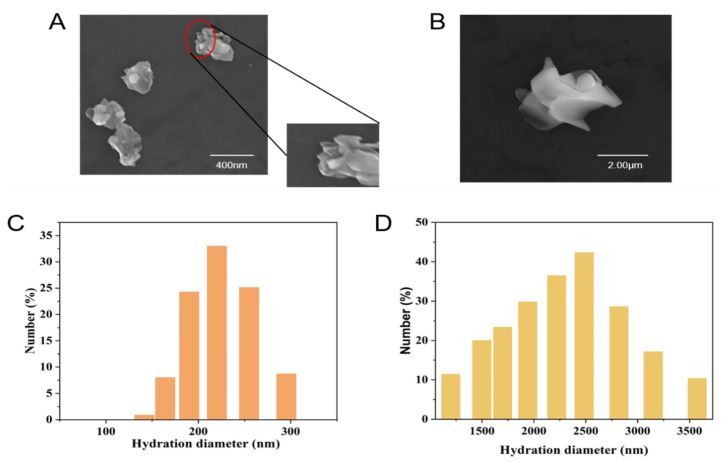
SEM images of (**A**) nano-WSe_2_ and (**B**) micro-WSe_2_ and DLS images of (**C**) nano-WSe_2_ and (**D**) micro-WSe_2_.

The SEM images for nano-WSe_2_ and micro-WSe_2_ is shown in Figure 1A,B. The size of nano-WSe_2_ is around 150 nm and it is around 2.5 μm for micro-WSe_2_. The particle size of nano-WSe_2_ through DLS is between 200–300 nm, and the particle size of micro-WSe_2_ is 1–3 μm (Figure 1C,D). It can be seen that both nano-WSe_2_ and micro-WSe_2_ are in the form of flakes, stacked layer by layer, which is consistent with other studies [13].

### 2.2. The Impacts on the Germination and Growth of Rice Plants by Nano-WSe_2_ or Micro-WSe_2_

#### 2.2.1. The Impacts on Germination Rate of Rice Seeds

The germination rate when exposed to different concentrations of nano-WSe_2_ or micro-WSe_2_ is shown in Table 1. It can be seen both nano-WSe_2_ and micro-WSe_2_ had no significant difference on the germination rate of rice seeds under the studied concentrations up to 5000 mg/L. On the other hand, compared with the control group, the germination rate of seeds exposed to different concentrations both nano-WSe_2_ and micro-WSe_2_ was lowered, although not significantly different, suggesting a potential inhibition on the germination of rice seeds.

#### 2.2.2. The Impacts on the Growth of Rice Seedlings

The lengths of roots, stems, and leaves in different groups are shown in Table 2. At the same concentration, the root length in nano-WSe_2_ group was shorter than that in the micro-WSe_2_ group. With increased concentration, the root length of rice exposed to nano-WSe_2_ became even shorter, especially at the concentration of 1000 and 5000 mg/L, and the root growth inhibition rate reached 30.94% and 66.67%, respectively, suggesting higher toxicity to roots by nano-WSe_2_ than micro-WSe_2_. Roots are an important site for the absorption, synthesis, and secretion of physiologically active substances required by rice [28]. In the nano-WSe_2_ group, it was found that the growth of the root system was restricted, which affected the growth and development of rice. When considering the impacts on the lengths of stems and leaves of rice, it can be seen that both nano-WSe_2_ and micro-WSe_2_ exposure generally led to decreased lengths, although not significantly different.

### 2.3. The Concentration of Se and W in Rice Plants after Exposure to Nano-WSe_2_ or Micro-WSe_2_

**Figure 2:**
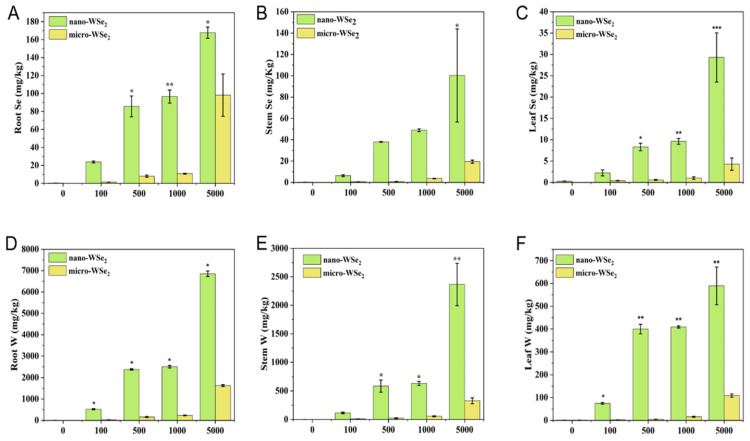
Concentration of Se (**A**–**C**) and W (**D**–**F**) in rice roots, stems, and leaves after exposure to different concentrations of nano-WSe_2_ or micro-WSe_2_ (* *p <* 0.5, ** *p <* 0.05, *** *p* < 0.01, *n* = 3).

The concentration of Se and W in the roots, stems, and leaves of rice is shown in Figure 2. The content of Se and W in rice exposed to nano-WSe_2_ was significantly higher than that of micro-WSe_2_ at the same concentration, which indicated that nano-WSe_2_ was more easily absorbed by rice and transported to other parts of rice than micro-WSe_2_. On the other hand, the accumulation of Se and W was dose-dependent since higher concentration exposure led to higher Se and W levels in rice tissues. Studies have shown that the concentration of accumulated Se in most plants was lower than 100 mg/kg [29] and our results agree with this. Studies have found that W in plants growing in the W mine area can reach 1637 mg/kg or even 13,500 mg/kg [30], which is much higher than what we observed in this study. Moreover, the concentration of Se and W in rice was in the order of roots > stems > leaves, suggesting roots are the major accumulation sites in rice. The high accumulation of Se and W in roots may explain the shortened root length as previously mentioned (Table 2). The molar ratio of Se to W in different tissues of rice was calculated and is shown in Table 3. It can be seen that the molar ratio of Se to W in rice tissues was lower than the control and much less than 2 in all the tissues of rice due to the high accumulation of W in rice tissues.

### 2.4. The Levels of GSH-Px an MDA in Rice Roots, Stems, and Leaves after Exposure to by Nano-WSe_2_ or Micro-WSe_2_

In higher plants, Glutathione peroxidase (GSH-Px) is a sulfur-containing peroxidase that can scavenge H_2_O_2_, organic hydroperoxides, and lipid peroxides in the body and block ROS from causing further damage to the body [31]. Therefore, the GSH-Px activity can reflect the oxidative damage in plant. Moreover, Se is required for the synthesis of GSH-Px. Malondialdehyde (MDA) is the product of cell membrane lipid peroxidation, which can be used as an indicator for lipid peroxidation in plants [32].

**Figure 3:**
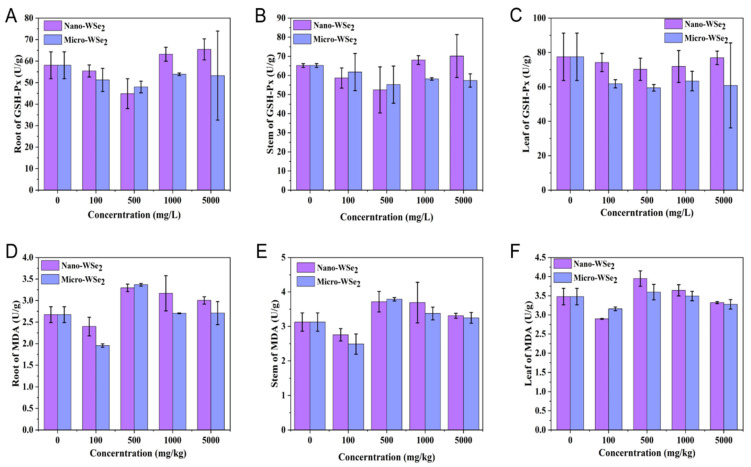
GSH-Px (**A**–**C**) and MDA (**D**–**F**) in rice roots, stems, and leaves after exposure to different concentrations of nano-WSe_2_ or micro-WSe_2_.

The levels of GSH-Px and MDA in roots, stems, and leaves are shown in Figure 3. From Figure 3A–C, no significant difference was found for GSH-Px levels in rice roots, stems, and leaves between the nano-WSe_2_ and micro-WSe_2_ groups. Many studies showed that Se can regulate the activity of antioxidant enzymes to a certain extent in higher plants such as wheat, rice, and cucumber [33]. It is known that Se is required for GSH-Px synthesis; however, no difference was found in this study when exposed to nano-WSe_2_ or micro-Wse_2_, suggesting that Se from nano-Wse_2_ or micro-Wse_2_ was not used for the formation of GSH-Px.

The levels of MDA in rice roots, stems, and leaves are shown in Figure 3D–F. It can be seen that the levels of MDA in rice cultivated by nano-WSe_2_ is higher than that in micro-WSe_2_, suggesting that nano-WSe_2_ brings more lipid peroxidation damage to rice than micro-WSe_2_. This agrees with the results that nano-WSe_2_ was more easily absorbed and transformed by rice than micro-WSe_2_.

Combined with the content of Se and W in rice and the values of GSH-Px and the level of MDA in rice, WSe_2_ was absorbed by rice and can be transported in rice, and there were peroxidative effects of WSe_2_ on rice. It was reported that the growth of plants was impaired when >3900 mg/kg in broccoli [21]. However, the concentration differences in hydroponic plants would be related to plant-specific tolerance mechanisms [34].

### 2.5. The Distribution and Transformation of Se and W in Rice Plants

#### 2.5.1. The Distribution of Se and W in Rice Plants

**Figure 4:**
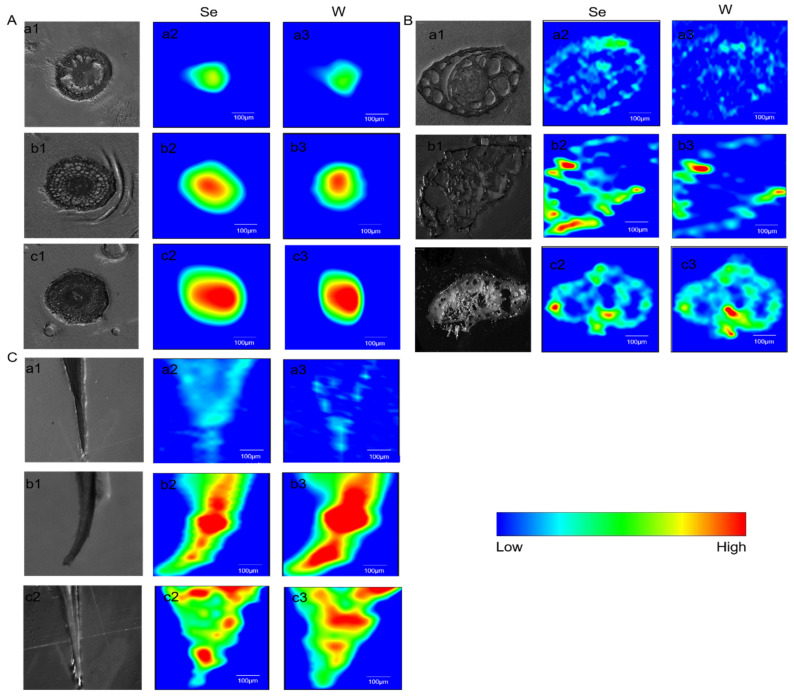
Spatial distribution of Se and W in rice (**A**) roots, (**B**) stems, and (**C**) leaves. (**a1**–**a3**) the control group; (**b1**–**b3**) the nano-WSe_2_ group; (**c1**–**c3**) the micro-WSe_2_ group.

SRXRF is a multi-element analysis and in-situ elemental analysis technique with the advantages of high sensitivity and high spatial resolution [35]. The spatial distribution of Se and W in rice roots, stems, and leaves is shown in Figure 4. It can be seen that W and Se distributed mostly in the stele of rice roots, suggesting the absorption and transportation of W and Se from rhizosphere to the stele. The W and Se were transported upward along the vascular bundle and distributed in the stems and leaves, and also distributed in the veins of the rice leaves. Furthermore, W and Se were found coexisting in the tissues of rice. Combined with the element content, as found through ICP-MS, this confirmed that nano-WSe_2_ was more easily absorbed by rice than micro-WSe_2_.

#### 2.5.2. The Transformation of Se and W in Rice Roots

**Figure 5:**
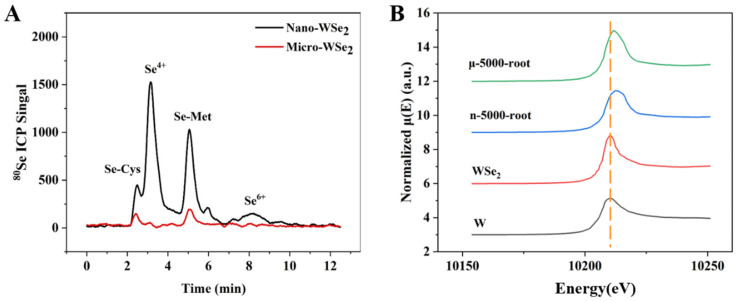
The species of Se and W in rice roots exposed to 5000 mg/L nano-WSe_2_ or micro-WSe_2_. (**A**) The speciation of Se by AE-HPLC-ICP-MS. (**B**) The chemical forms of W by XAS.

The species of Se and W in rice roots are shown in Figure 5. From Figure 5A, higher levels of Se-Cys, Se-Met, and Se^IV^ can be found in nano-WSe_2_ exposed rice roots, suggesting that nano-WSe_2_ is more easily transformed than micro-WSe_2_.

The oxidation state of tungsten in rice roots was studied by XAS. Figure 5B shows that the absorption edge of W in rice roots is right-shifted, suggesting that W has been oxidized to a higher valence than WSe_2_, most possibly to tungstate, which is a plant-available form [36]. This agrees with other studies that found W in rice was in the form of tungstate [37]. Studies have shown that the effects of tungstate on plants are bidirectional. For example, 1–50 mg/L tungstate in broccoli will not cause oxidative damage and is almost harmless, and it also has certain beneficial effects. At the dose of 100 mg/L, tungstate caused the reduction of germination rate, growth parameters, and some biochemical activities [36]. Moreover, studies have shown that low tungstate levels have a protective mechanism for the GSH-GSH-Px-GST system in plants and can maintain the amount of toxic reactive oxygen species and lipid peroxides under control plants [21].

These results suggested that WSe_2_ at the micro- and nano-scale could also be transformed by the rice plants, which raised the safety concerns about the application of TMDCs, such as nano-WSe_2_. However, it is still unclear how nano-WSe_2_ was transformed in these species, which deserves further study.

## 3. Materials and Methods

### 3.1. Reagents and Materials

WSe_2_ powder (99.9%) was purchased from Alfa Aesar (Shanghai, China), which was used as micro-WSe_2_. Nano-WSe_2_ was obtained from Xinchao New Materials Co. Ltd. (Rizhao, China). H_2_O_2_ (MOS) was obtained from Sinopharm (Shanghai, China). HNO_3_ (BV-III) was provided by Beijing Chemical Plants (Beijing, China). Sodium selenate (Na_2_SeO_4_), sodium selenite (Na_2_SeO_3_), selenocysteine (SeCys) and selenomethionine (SeMet) were used as standard for HPLC analysis, were commercially purchased from Sigma Aldrich (Merck KGaA, Darmstadt, Germany). Methanol was from Guangfu and ammonium citrate (98.5%) from Sigma. Deionized water was prepared by a Milli-Q system (Millipore, Bedford, MA, USA).

### 3.2. Characterization of the Micro-WSe_2_ and Nano-WSe_2_

The particle size was measured by a dynamic light scattering particle size analyzer (Zetasizer Nano ZS90, Malvern, UK) and Scanning Electron Microscope (SU8220, Tokyo, Japan).

### 3.3. Rice Germination and Hydroponic Cultivation

The germination of rice seeds follows Zhao et al. [38]. Briefly, the full-grained Y Liangyou 900 rice seeds were immersed in 30% hydrogen peroxide for 30 min for disinfection and then rinsed with deionized water to wash off the disinfectant followed by soaking in deionized water for 2 h. Fifty soaked seeds were placed into each petri dish with 5 mL nano-WSe_2_ or micro-WSe_2_ (0, 100, 500, 1000, or 5000 mg/L) with triplicate dishes in each dose. Previous studies showed that the cell death rate at 400 mg/L was 50% for nano-WSe_2_ [15]. Since the culture state of plants and cells is different, the high concentration up to 5000 mg/L was used in this study was higher than the previous used. The seeds were put in an artificial climate chamber at a temperature of 27 °C, light intensity of 0 (dark conditions), and humidity of between 50–70%. The germination rate of the seeds in each petri dish was counted on the 7th day.

The rice seedlings were then cultured with 5 mL nano-WSe_2_ or micro-WSe_2_ (0, 100, 500, 1000, or 5000 mg/L) in an artificial climate chamber. Hoagland’s solution was added at a fixed time each day. The daytime temperature was set at 27 °C with a light intensity of 300–350 μmol·m^−2^·s^−1^ for 14 h; the nighttime temperature was set at 20 °C with no light for 10 h. The relative humidity was controlled at 50–70%. On the 30th day, the rice plants were harvested and cleaned thoroughly using deionized water and kept in the refrigerator prior to further analysis. The lengths of roots, stems, and leaves were recorded in each group.

### 3.4. Analytical Method

#### 3.4.1. Quantification of W and Se in Rice Plants

The concentration of W and Se in rice tissues was determined using an inductively coupled plasma-mass spectrometry (ICP-MS) (Thermo Elemental X7, Waltham, MA, USA) [39]. Each experiment was performed in triplicate. Citrus leaves (GBW10020/GSB-11) were used as the certified reference material, and the recovery rate of Se was 83%, while the recovery rate of W was 97% when using the tungsten standard solution.

#### 3.4.2. Measurement of GSH-Px and MDA

The activity of glutathione peroxidase (GSH-Px) and the levels of malonaldehyde (MDA) in plant tissues were determined by a multi-function microplate reader (Infinite M200 Pro, TECAN Trading AG, Switzerland) using commercial assay kits (Nanjing Jian cheng Bioengineering Institute, Nanjing, China). Each experiment was performed in triplicate.

#### 3.4.3. The Speciation of Se in Rice Tissues

Anion Exchange High Performance Liquid Chromatography Combined with Inductively Coupled Plasma Mass Spectrometry (AE-HPLC-ICP-MS) was used to analyze Se species in rice tissues [40]. A Hamilton PRP-Xl00 (250 mm × 4, 1 mm, 10 mm) was used for the separation of Se species [41]. The mobile solution was as follows: 0.5 mM ammonium citrate solution + 2% (*v*/*v*) methanol, pH 3.7, as mobile phase A and 100 mM ammonium citrate solution + 2% (*v*/*v*) methanol as mobile phase B with gradient elution conditions of 93% A + 7% B.

Plant samples were processed by enzymatic hydrolysis: 20 mg of root samples were weighed into a 15 mL centrifuge tube and 6 mg protease, 4 mg lipase, and 1 mL Tris-HCl (pH = 7.5) were added. The samples were shaken in a water bath shaker at 200 rpm for 24 h at 37 °C and were then centrifuged at 4000 rpm and 15 °C for 15 min; the supernatant was then collected after centrifugation. The above steps were repeated, and the supernatants obtained by centrifugation were mixed together. The supernatants were filtered with a 0.22 μm microporous membrane.

#### 3.4.4. The Distribution and Transformation of W and Se in Rice Tissues

Synchrotron radiation X-ray fluorescence (SR-XRF) was used to study the distribution of W and Se in rice tissues. It was performed at beamline 4W1B, Beijing synchrotron Radiation Facility, which runs at the energy of 2.5 GeV with a current intensity of 150–250 mA. A polychromatic beam (pink beam) with the energy of 10–18 keV was used as the incident X-ray. The mapping was performed with a beam size of 50 × 50 µm^2^. A four-element Hitachi Vortex^®^-ME4 silicon drift detector coupled to a Quantum Detectors Xspress3 multi-channel analyzer system was used [42]. Spectral data were normalized by PyMCA [43] (Python multichannel analyzer) and then plotted by Origin 9.0 (OriginLab).

An X-ray absorption spectroscopy (XAS) experiment was performed at beamline 4B9A, Beijing synchrotron Radiation Facility. Elemental W and WSe_2_ were used as standard references. IFEFFIT Athena software (CARS, the Consortium for Advanced Radiation Sources at University of Chicago) was used for data analysis [44].

### 3.5. Statistical Analysis

Statistical tests were performed using the software Origin 9.0, and *p* < 0.05 was considered to be significant. All data were presented as mean ± standard deviations, and all data were analyzed using one-way ANOVA. Student’s *t*-test was used to compare means among groups.

## 4. Conclusions

The wide application of TMDCs leads to the increased possibility of their release into the environment. This study found that nano-WSe_2_ did not affect the germination of rice seeds up to 5000 mg/L but affected the growth of rice seedlings, especially at higher concentrations. Nano-WSe_2_ was much more easily absorbed and transported to other parts of rice than micro-WSe_2_. The high content of W and Se was found in roots than that in stems and leaves, suggesting most nano-WSe_2_ were blocked by roots. HPLC-ICP-MS and XAS found that nano-WSe_2_ was transformable by rice plants. In all, our results raised the safety concerns about the application of TMDCs such as nano-WSe_2_. However, it is still unclear how nano-WSe_2_ and micro-WSe_2_ were transformed in the plants, which deserves further study. In addition, the impacts on the growth of rice throughout its life cycle and whether the soil microbiota will be affected by nano-WSe_2_ have not been investigated. Therefore, further studies on the long-term effects of WSe_2_ on plants are also warranted.

## Figures and Tables

**Table 1:** The germination rate of rice seeds on the 7th day.

Concentration (mg/L)	Germination Rate (%)
0	90.66 ± 0.01
(nano) 100	81.33 ± 0.01
(micro) 100	82.00 ± 0.07
(nano) 500	85.33 ± 0.02
(micro) 500	87.86 ± 0.07
(nano) 1000	81.33 ± 0.06
(micro) 1000	87.33 ± 0.03
(nano) 5000	86.67 ± 0.05
(micro) 5000	83.33 ± 0.03

**Table 2:** The length of roots, stems, and leaves of rice plants on the 30th day when exposed to different concentrations of nano-WSe_2_ or micro-WSe_2_ (* *p* < 0.05, ** *p* < 0.0001).

Concentration (mg/L)	The Length of Root (cm)	The Length of Stem (cm)	The Length of Leaf (cm)
0	6.69 ± 0.22	4.46 ± 1.22	8.59 ± 0.25
(nano) 100	6.22 ± 0.28	4.13 ± 0.16	8.62 ± 0.71
(micro) 100	6.30 ± 0.01	3.52 ± 0.28	8.21 ± 0.20
(nano) 500	5.79 ± 0.52	3.60 ± 0.31	9.45 ± 0.21
(micro) 500	7.17 ± 1.06	3.39 ± 0.04	7.78 ± 0.11
(nano) 1000	4.62 ± 0.22 *	4.09 ± 0.32	8.47 ± 0.59
(micro) 1000	6.93 ± 0.16	3.34 ± 0.21	7.77 ± 0.55
(nano) 5000	2.23 ± 0.49 **	3.54 ± 0.17	8.39 ± 0.31
(micro) 5000	5.97 ± 0.20	3.42 ± 0.02	6.76 ± 0.11

**Table 3:** Molar ratio of selenium and tungsten (n_Se_:n_W_) in rice.

Concentration (mg/kg)	Root	Stem	Leaf
0	0.14	0.21	0.80
(nano) 100	0.11	0.13	0.07
(micro) 100	0.11	0.14	0.40
(nano) 500	0.08	0.14	0.05
(micro) 500	0.12	0.10	0.18
(nano) 1000	0.06	0.07	0.05
(micro) 1000	0.11	0.14	0.15
(nano) 5000	0.06	0.11	0.09
(micro) 5000	0.14	0.14	0.09

## Data Availability

Not applicable.

## Sample Availability

Samples of the compounds are available from the retailers and the authors.

## References

[1] Nair N.L., Maniv E., John C., Doyle S., Orenstein J., Analytis J.G. (2020). Electrical switching in a magnetically intercalated transition metal dichalcogenide. Nat. Mater..

[2] Shi Z.-T., Kang W., Xu J., Sun Y.-W., Jiang M., Ng T.-W., Xue H.-T., Yu D.Y.W., Zhang W., Lee C.-S. (2016). Hierarchical nanotubes assembled from MoS2-carbon monolayer sandwiched superstructure nanosheets for high-performance sodium ion batteries. Nano Engry.

[3] Gao Y., Chen C., Tan X., Xu H., Zhu K. (2016). Polyaniline-modified 3D-flower-like molybdenum disulfide composite for efficient adsorption/photocatalytic reduction of Cr(VI). J. Colloid Interface Sci..

[4] Yin W., Yu J., Lv F., Yan L., Zheng L.R., Gu Z., Zhao Y. (2016). Functionalized Nano-MoS_2_ with Peroxidase Catalytic and Near-Infrared Photothermal Activities for Safe and Synergetic Wound Antibacterial Applications. ACS Nano.

[5] Zhao L., Lu L., Wang A., Zhang H., Huang M., Wu H., Xing B., Wang Z., Ji R. (2020). Nano-Biotechnology in Agriculture: Use of Nanomaterials to Promote Plant Growth and Stress Tolerance. J. Agric. Food Chem..

[6] Zhao L., Chen S., Tan X., Yan X., Zhang W., Huang Y., Ji R., White J.C. (2022). Environmental implications of MoS_2_ nanosheets on rice and associated soil microbial communities. Chemosphere.

[7] Sarkar D., Liu W., Xie X., Anselmo A.C., Mitragotri S., Banerjee K. (2014). MoS2 Field-Effect Transistor for Next-Generation Label-Free Biosensors. ACS Nano.

[8] Cao M., Cai R., Zhao L., Guo M., Wang L., Wang Y., Zhang L., Wang X., Yao H., Xie C. (2021). Molybdenum derived from nanomaterials incorporates into molybdenum enzymes and affects their activities in vivo. Nat. Nanotechnol..

[9] Li Y., Jin Q., Yang D., Cui J. (2018). Molybdenum Sulfide Induce Growth Enhancement Effect of Rice (*Oryza sativa* L.) through Regulating the Synthesis of Chlorophyll and the Expression of Aquaporin Gene. J. Agric. Food Chem..

[10] Wen B., Zhu Y., Yudistira D., Boes A., Zhang L., Yidirim T., Liu B., Yan H., Sun X., Zhou Y. (2019). Ferroelectric-Driven Exciton and Trion Modulation in Monolayer Molybdenum and Tungsten Diselenides. ACS Nano.

[11] Bertolazzi S., Gobbi M., Zhao Y., Backes C., Samorì P. (2018). Molecular chemistry approaches for tuning the properties of two-dimensional transition metal dichalcogenides. Chem. Soc. Rev..

[12] Huang Y., Zhao Y., Liu Y., Ye R., Chen L., Bai G., Xu S. (2021). Erbium-doped tungsten selenide nanosheets with near-infrared II emission and photothermal conversion. Chem. Eng. J..

[13] Wang W., Gu W., Li G., Xie H., Wong P.K., An T. (2020). Few-layered tungsten selenide as a co-catalyst for visible-light-driven photocatalytic production of hydrogen peroxide for bacterial inactivation. Environ. Sci. Nano.

[14] Lin Y.T., Zhang X.Q., Chen P.H., Chi C.C., Lin E.C., Rong J.G., Ouyang C., Chen Y.F., Lee Y.H. (2020). Selective Growth of WSe_2_ with Graphene Contacts. Nanoscale Res. Lett..

[15] Teo W.Z., Chng E.L., Sofer Z., Pumera M. (2014). Cytotoxicity of exfoliated transition-metal dichalcogenides (MoS_2_, WS_2_, and WSe_2_) is lower than that of graphene and its analogues. Chemistry.

[16] Pushie M.J., George G.N. (2011). Spectroscopic studies of molybdenum and tungsten enzymes. Coord. Chem. Rev..

[17] Bevers L.E., Hagedoorn P.-L., Hagen W.R. (2009). The bioinorganic chemistry of tungsten. Coord. Chem. Rev..

[18] Seelmann C.S., Willistein M., Heider J., Boll M. (2020). Tungstoenzymes: Occurrence, Catalytic Diversity and Cofactor Synthesis. Inorganics.

[19] Zhu W., Winter M.G., Byndloss M.X., Spiga L., Duerkop B.A., Hughes E.R., Buttner L., de Lima Romao E., Behrendt C.L., Lopez C.A. (2018). Precision editing of the gut microbiota ameliorates colitis. Nature.

[20] Kumar A., Aery N.C. (2011). Effect of tungsten on growth, biochemical constituents, molybdenum and tungsten contents in wheat. Plant Soil Environ..

[21] Dawood M.F.A., Azooz M.M. (2020). Insights into the oxidative status and antioxidative responses of germinating broccoli (*Brassica oleracea* var. *italica* L.) seeds in tungstate contaminated water. Chemosphere.

[22] White P.J. (2018). Selenium metabolism in plants. Biochim. Biophys. Acta Gen. Subj..

[23] Dimkovikj A., Fisher B., Hutchison K., Van Hoewyk D. (2015). Stuck between a ROS and a hard place: Analysis of the ubiquitin proteasome pathway in selenocysteine treated Brassica napus reveals different toxicities during selenium assimilation. J. Plant Physiol..

[24] Li L., Liu Z., Quan J., Lu J., Zhao G., Sun J. (2022). Metabonomics analysis reveals the protective effect of nanoselenium against heat stress of rainbow trout (*Oncorhynchus mykiss*). J. Proteom..

[25] Liu F., Celi P., Cottrell J.J., Chauhan S.S., Leury B.J., Dunshea F.R. (2018). Effects of a short-term supranutritional selenium supplementation on redox balance, physiology and insulin-related metabolism in heat-stressed pigs. J. Anim. Physiol. Anim. Nutr..

[26] Yuanan H., He K., Sun Z., Chen G., Cheng H. (2020). Quantitative source apportionment of heavy metal(loid)s in the agricultural soils of an industrializing region and associated model uncertainty. J. Hazard. Mater..

[27] Li Z., Liang Y., Hu H., Shaheen S.M., Zhong H., Tack F.M.G., Wu M., Li Y.F., Gao Y., Rinklebe J. (2021). Speciation, transportation, and pathways of cadmium in soil-rice systems: A review on the environmental implications and remediation approaches for food safety. Environ. Int..

[28] Yang J.-c., Zhang H., Zhang J.-h. (2012). Root Morphology and Physiology in Relation to the Yield Formation of Rice. J. Integr. Agric..

[29] El-Ramady H., Abdalla N., Taha H.S., Alshaal T., El-Henawy A., Faizy S.E.D.A., Shams M.S., Youssef S.M., Shalaby T., Bayoumi Y. (2015). Selenium and nano-selenium in plant nutrition. Environ. Chem. Lett..

[30] Strigul N., Koutsospyros A., Arienti P., Christodoulatos C., Dermatas D., Braida W. (2005). Effects of tungsten on environmental systems. Chemosphere.

[31] Niwa Y., Sasaki Y. (2003). Plant self-defense mechanisms against oxidative injury and protection of the forest by planting trees of triploids and tetraploids. Ecotoxicol. Environ. Saf..

[32] Liu S., Waqas M.A., Wang S.H., Xiong X.Y., Wan Y.F. (2017). Effects of increased levels of atmospheric CO_2_ and high temperatures on rice growth and quality. PLoS ONE.

[33] Xu H., Yan J., Qin Y., Xu J., Shohag M.J.I., Wei Y., Gu M. (2020). Effect of Different Forms of Selenium on the Physiological Response and the Cadmium Uptake by Rice under Cadmium Stress. Int. J. Environ. Res. Public Health.

[34] Kennedy A.J., Johnson D.R., Seiter J.M., Lindsay J.H., Boyd R.E., Bednar A.J., Allison P.G. (2012). Tungsten toxicity, bioaccumulation, and compartmentalization into organisms representing two trophic levels. Environ. Sci. Technol..

[35] Gao Y., Liu N., Chen C., Luo Y., Li Y., Zhang Z., Zhao Y., Zhao B., Iida A., Chai Z. (2008). Mapping technique for biodistribution of elements in a model organism, Caenorhabditis elegans, after exposure to copper nanoparticles with microbeam synchrotron radiation X-ray fluorescence. J. Anal. At. Spectrom..

[36] Dawood M.F.A., Azooz M.M. (2019). Concentration-dependent effects of tungstate on germination, growth, lignification-related enzymes, antioxidants, and reactive oxygen species in broccoli (*Brassica oleracea* var. italica L.). Environ. Sci. Pollut. Res. Int..

[37] Shi N., Bai T., Wang X., Tang Y., Wang C., Zhao L. (2022). Toxicological effects of WS_2_ nanomaterials on rice plants and associated soil microbes. Sci. Total Environ..

[38] Zhao J., Li Y., Li Y., Gao Y., Li B., Hu Y., Zhao Y., Chai Z. (2014). Selenium modulates mercury uptake and distribution in rice (*Oryza sativa* L.), in correlation with mercury species and exposure level. Metallomics.

[39] Pan M.Y., Zang Y., Zhou X.R., Lu Y.L., Xiong J.P., Li H.M., Feng L.X. (2021). Inductively coupled plasma mass spectrometry for metrometallomics: The study of quantitative metalloproteins. At. Spectrosc..

[40] Li H. (2021). Clinimetallomics: Arsenic Speciation in Urine from Patients with Arsenism by HPLC-ICP-MS. At. Spectrosc..

[41] Hu L., Dong Z., Huang X., Li Y.F., Li B., Qu L., Wang G., Gao Y., Chen C. (2011). Determination of selenium chemical species in serum after selenium intervention in people with long-term mercury exposure by anion exchange chromatography inductively coupled plasma mass spectrometry. Chin. J. Anal. Chem..

[42] He L., Lu Y., Li C., Xie H., Zhao J., Wang Y., Wang L., Wang X., Wang W., Chen D. (2022). Non-targeted metallomics through synchrotron radiation X-ray fluorescence with machine learning for cancer screening using blood samples. Talanta.

[43] Solé V.A., Papillon E., Cotte M., Walter P., Susini J. (2007). A multiplatform code for the analysis of energy-dispersive X-ray fluorescence spectra. Spectrochim. Acta Part B At. Spectrosc..

[44] Li Z.J., Huang Z.W., Guo W.L., Wang L., Zheng L.R., Chai Z.F., Shi W.Q. (2017). Enhanced Photocatalytic Removal of Uranium(VI) from Aqueous Solution by Magnetic TiO_2_/Fe_3_O_4_ and Its Graphene Composite. Environ. Sci. Technol..

